# Tackling the scaling-up problem of digital health applications

**DOI:** 10.1007/s10389-021-01599-7

**Published:** 2021-06-05

**Authors:** Peggy Richter, Lorenz Harst

**Affiliations:** 1 Faculty of Business and Economics, Chair of Wirtschaftsinformatik, esp. Systems Development, Technische Universität Dresden, Dresden, Germany; 2 Center for Evidence-based Healthcare, University Hospital and Faculty of Medicine Carl Gustav Carus, Technische Universität Dresden, Fetscherstraße 747, 01307 Dresden, Germany

**Keywords:** Digital health, Implementation, Evaluation, Pandemic management

## Abstract

**Aim:**

The purpose of this editorial is to provide guidance for the readers concerning the broad realm of approaches towards successful implementation of digital health applications into the health care system. Recent developments due to the challenges posed by the COVID-19 pandemic are used as a current angle.

**Subject and Methods:**

All contributions within the special issue were scanned for their most decisive contribution to the special issue and the field of implementation science, with a focus on digital health. Micro, meso, and macro layers of implementation processes, as well as the technological perspective itself, are used as broad categories for sorting the contributions and structuring the special issue.

**Results:**

The ten contributions to this special issue cover micro (*n* = 1), technology (*n* = 1), meso (*n* = 4) and macro (*n* = 2) perspectives on the implementation process of digital health applications. Two further contributions also tackle the issue from a wider perspective when aiming to structure telemedicine application types and barriers encountered when implementing digital health.

**Conclusion:**

Considering the wide array of research fields represented in this special issue, an emphasis is put on the importance of interdisciplinary work required for tackling the scale-up problem of digital health. As such, the special issue can assist in leveraging the full potential of digital health, not only when dealing with situations as out-of-the-ordinary as the current pandemic but also well beyond that, for example when dealing with the upcoming challenges of demographic change.

## Editorial to this special issue

The COVID-19 pandemic has not only changed our daily lives, but has also brought changes in the way health care is delivered. Digital health applications are increasingly used in order to deliver care not only to those who suffer from COVID-19 but also to the chronically ill and to those afflicted with acute ailments (World Health Organisation (WHO) 2015). At the same time, using digital health applications helps to protect health care personnel and other patients from an infection with the virus (Bashshur et al. 2020).

This pandemic is pushing health care to new frontiers and is at the same time questioning traditional ways in which services are delivered. Deadlocked barriers for a successful implementation of digital health solutions were suddenly torn down to quickly exploit the positive effects of digital health (for a quick overview of such initiatives, see Anthony 2020).

In this special issue, we bundle current findings with regard to strategies for successfully and sustainably implementing digital health solutions while considering barriers on four different levels. These levels are related to (I) the users of digital health solutions, i.e. individual patients or health care professionals in their working environment (micro level), (II) their respective wider societal structure (meso level), (III) the regulatory or financial framework of the health care system (macro level), or (IV) the technology to be implemented. A broad set of implementation barriers is presented in the systematic work by Gleiß and Lewandowksi in this special issue.

On the macro level, regulations and laws long thought to be barriers for successfully implementing digital health applications were broken down quickly, e.g. considerations of data security and financing (Anthony 2020). Thus, the caregiver community was enabled to rely on digital health in their line of work, mostly because alternatives were lacking (Elson et al. 2020). Fittingly, core readiness and other factors improving community readiness for telemedicine, as one phenotype of digital health (Otto et al. 2020), are analyzed from the perspective of historic projects in the article by Reifegerste and colleagues, while the important topic of privacy regulation is picked up on by Jusob and colleagues in this special issue. The former contribution illuminates the importance of all stakeholders having a common understanding of remote health care delivery being the only valid solution to a certain gap in health care delivery. The latter contribution aims at building a sustainable mHealth privacy framework meant to overcome prevalent privacy concerns by building on reviewing existing regulatory standards.

Still, even in times of pressing need as we face it during this pandemic, unsolved problems remain, preventing the widespread and long-term use of digital health applications. Among them is the issue of connectivity on the level of the technology itself, which might be low in the Western hemisphere (Bashshur et al. 2020), yet remains challenging for developing countries (Anthony 2020) and extremely remote areas as is discussed in the article by Mastella and colleagues. The authors demonstrate the challenges posed on telemedicine-aided health care delivery to offshore wind parks, with low internet connectivity being a prominent one.

From previous SARS outbreaks we know that interoperability, especially of hospital information systems on the meso level, has been a pitfall when it comes to sharing data between health care providers — a necessary process for monitoring active cases as well as chronically ill patients (Huang et al. 2017). Learning from the example of Health Smart Homes, where a common “language” is the basis of successful data sharing (Wollschlaeger and Kabitzsch 2019), might help with interoperability issues such as the ones described in the articles by Müller and colleagues included in this special issue. In their contribution, the authors give insights into the development of a multi-centered data sharing platform for cardiovascular diseases and its extension to COVID-19 cases.

Even though health care providers (on the micro level) are increasingly willing to use telemedicine in pandemic times (Bashshur et al. 2020), worries concerning the loss of face-to-face contact in medical consultations remain with patients and providers alike (Iyengar et al. 2020). Therefore, it is essential to take user perspectives into account in the development process of any digital health application (Harst et al. 2019). The article by Brauner and Ziefle included in this special issue addresses this aspect in the domain of gamified applications, demonstrating the user-centered development of digital games for the management of chronic diseases.

In general, successful implementation of digital health application into existing systems (meso level) is an outcome which is not sufficiently considered when designing evaluation studies for digital health applications (National Institute for Health and Care Excellence 2019). This makes implementation strategies and their evaluation an important future research need in the field of digital health (Timpel and Harst 2020). In this special issue, Fürstenau and colleagues demonstrate challenges when implementing a digitally aided value-based health care approach to frailty assessment in a hospital as well as means to overcome them. Greve and colleagues address the topic of implementing mHealth solutions in low-resource environments, and provide strategies based on expert interviews. The challenging issue of implementing digital health applications for the remote management of health care issues in existing care structures during the COVID-19 pandemic is addressed in the work by Balagna and colleagues.

On a more basal level, the successful development and evaluation of digital health applications is based on a common nomenclature and phenotyping of application types, as proposed by the taxonomy developed by Harst and colleagues.

All in all, this special issue tackles the persisting scaling-up problem for digital health applications in an interdisciplinary manner — including contributions from the field of cardiology, evidence-based medicine, economics, and health communication. Given the variety of applications and broader solutions proposed and discussed, the special issue also reflects the heterogeneity in the field of digital health. Taking into account not only short-term challenges to the health care system as represented by the COVID-19 pandemic but also the progression of demographic change and the rise in health care demands it entails, digital health is one piece in the complex puzzle of providing adequate health care for all (Eysenbach 2001). With this special issue, propositions are made as to how to speed up this process.

## Data Availability

N/A
